# Small molecule inhibitors of hnRNPA2B1–RNA interactions reveal a predictable sorting of RNA subsets into extracellular vesicles

**DOI:** 10.1093/nar/gkaf176

**Published:** 2025-03-18

**Authors:** Jessica Corsi, Pouriya Sharbatian Semnani, Daniele Peroni, Romina Belli, Alessia Morelli, Michelangelo Lassandro, Viktoryia Sidarovich, Valentina Adami, Chiara Valentini, Paolo Cavallerio, Julian Grosskreutz, Fabrizio Fabbiano, Dajana Grossmann, Andreas Hermann, Gianluca Tell, Manuela Basso, Vito G D’Agostino

**Affiliations:** Laboratory of Biotechnology and Nanomedicine, Department of Cellular, Computational and Integrative Biology (CIBIO), University of Trento, Via Sommarive 9, 38123 Trento, Italy; Laboratory of Biotechnology and Nanomedicine, Department of Cellular, Computational and Integrative Biology (CIBIO), University of Trento, Via Sommarive 9, 38123 Trento, Italy; MS Core Facility, Department of Cellular, Computational and Integrative Biology (CIBIO), University of Trento, Via Sommarive 9, 38123 Trento, Italy; MS Core Facility, Department of Cellular, Computational and Integrative Biology (CIBIO), University of Trento, Via Sommarive 9, 38123 Trento, Italy; Laboratory of Biotechnology and Nanomedicine, Department of Cellular, Computational and Integrative Biology (CIBIO), University of Trento, Via Sommarive 9, 38123 Trento, Italy; Laboratory of Biotechnology and Nanomedicine, Department of Cellular, Computational and Integrative Biology (CIBIO), University of Trento, Via Sommarive 9, 38123 Trento, Italy; HTS Core Facility, Department of Cellular, Computational and Integrative Biology (CIBIO), University of Trento, Via Sommarive 9, 38123 Trento, Italy; HTS Core Facility, Department of Cellular, Computational and Integrative Biology (CIBIO), University of Trento, Via Sommarive 9, 38123 Trento, Italy; NGS Core Facility, Department of Cellular, Computational and Integrative Biomedicine CIBIO, University of Trento, 38122 Trento, Italy; NGS Core Facility, Department of Cellular, Computational and Integrative Biomedicine CIBIO, University of Trento, 38122 Trento, Italy; Excellence Cluster Precision Medicine in Inflammation, University of Lübeck, Ratzeburger Allee 160, 23538 Lübeck, Germany; Laboratory of Biotechnology and Nanomedicine, Department of Cellular, Computational and Integrative Biology (CIBIO), University of Trento, Via Sommarive 9, 38123 Trento, Italy; Translational Neurodegeneration Section “Albrecht Kossel”, Department of Neurology, University Medical Center Rostock, University of Rostock, 18147 Rostock, Germany; Translational Neurodegeneration Section “Albrecht Kossel”, Department of Neurology, University Medical Center Rostock, University of Rostock, 18147 Rostock, Germany; Center for Transdisciplinary Neurosciences Rostock (CTNR), University Medical Center Rostock, University of Rostock, 18147 Rostock, Germany; Deutsches Zentrum für Neurodegenerative Erkrankungen (DZNE) Rostock/Greifswald, 18147 Rostock, Germany; Laboratory of Molecular Biology and DNA repair, Department of Medicine (DMED), University of Udine, Piazzale M. Kolbe 4, 33100 Udine, Italy; Laboratory of Transcriptional Neurobiology, Department of Cellular, Computational and Integrative Biology (CIBIO), University of Trento, Via Sommarive 9, 38123 Trento, Italy; Laboratory of Biotechnology and Nanomedicine, Department of Cellular, Computational and Integrative Biology (CIBIO), University of Trento, Via Sommarive 9, 38123 Trento, Italy

## Abstract

Extracellular vesicles (EVs) are cell-secreted membranous particles contributing to intercellular communication. Coding and noncoding RNAs can be detected as EV cargo, and RNA-binding proteins (RBPs), such as hnRNPA2B1, have been circumstantially implicated in EV-RNA sorting mechanisms. However, the contribution of competitive RBP–RNA interactions responsible for RNA-sorting outcomes is still unclear, especially for predicting the EV-RNA content. We designed a reverse proteomic analysis exploiting the EV-RNA to identify intracellular protein binders *in vitro*. Using cells expressing a recombinant hnRNPA2B1 to normalize competitive interactions, we prioritized a network of heterogeneous nuclear ribonucleoproteins and purine-rich RNA sequences subsequently validated in secreted EV-RNA through short fluorescent RNA oligos. Then, we designed a GGGAG-enriched RNA probe that efficiently interacted with a full-length human hnRNPA2B1 protein. We exploited the interaction to conduct a pharmacological screening and identify inhibitors of the protein–RNA binding. Small molecules were orthogonally validated through biochemical and cell-based approaches. Selected drugs remarkably impacted secreted EV-RNAs and reduced an RNA-dependent, EV-mediated paracrine activation of NF-kB in recipient cells. These results demonstrate the relevance of post-transcriptional mechanisms for EV-RNA sorting and the possibility of predicting the EV-RNA quality for developing innovative strategies targeting discrete paracrine functions.

## Graphical Abstract

**Graphical Abstract:**
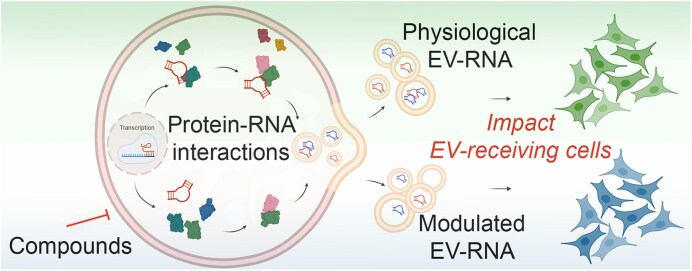

## Introduction

RNA-binding proteins (RBPs) orchestrate different RNA processing activities, from transcription, splicing, maturation, transport, and translation to RNA degradation. Recently, the secreted RNA fraction is increasingly recognized as a functional part of the conditional microenvironment in cancer and neurodegenerative diseases [1–5], being cargo of extracellular vesicles (EVs) [6, 7] mediating intercellular communication [8, 9]. EVs are cell-secreted membranous particles presenting size and molecular diversity due to heterogeneous protein, nucleic acid, and metabolic cargoes [10]. According to their biogenesis, EVs are classified as exosomes, ectosomes, and apoptotic bodies. Exosomes are typically less than 200 nm and generate as intraluminal vesicles within endosomes, involving endosomal sorting complex required for transport through-dependent or -independent mechanisms [11]. Ectosomes range from 100 to 1000 nm in diameter and derive from the outward budding and fission of the plasma membrane through mechanisms likely involving calcium and the interplay of ATP/GTP-dependent proteins [10]. Conversely, apoptotic bodies are larger particles formed during apoptosis [12].

Coding and noncoding RNAs can be detected in secreted EVs [13, 14]. However, the intracellular mechanisms of RNA selection for vesicular secretion are still unclear. Competing RBPs could significantly contribute to selecting EV transcripts, including, for example, microRNAs (miRNAs) [15], demonstrating that selective packaging can occur at the intracellular level for a desired secretion. We recently highlighted that heterogeneous nuclear ribonucleoproteins (hnRNPs) are frequently detected as vesicular protein cargoes [6]. Among them, TDP43, FUS, and hnRNPA2B1 represent molecular hallmarks of amyotrophic lateral sclerosis (ALS), where these RBPs are known for nuclear loss of function, aggregation tendency, and stress granules assembly [16–18]. In association with the diversity and function of bound RNAs, dosage alterations of several hnRNPs were also associated with cancer progression and autoimmune diseases [19–21].

Interestingly, hnRNPA2B1 was recently described as a crucial factor involved in the vesicular sorting of miRNAs through the recognition of specific EXO motifs (5′-AGG, 5′-UAG, 5′-GGAG) [22–24], providing circumstantial evidence that post-transcriptional mechanisms can mediate RNA selection and enrichment into secreted EVs. However, the quantitative contribution of competitive RBP–RNA interactions responsible for RNA-sorting outcomes still needs to be deciphered, especially for EV-RNA quantitation, manipulation, and predictability. To start addressing these biological questions, we designed a reverse proteomic strategy tailored to the secreted EV-RNA and “retrospective” protein binders *in vitro*. We found a significant enrichment of hnRNPs, including hnRNPA2B1, presenting several interaction nodes with RAB proteins already implicated in vesicular trafficking. We distinguished purine-rich RNA sequences as a common substrate recognized by the ranked hnRNPs and constituting a subset of previously identified EXO-motifs. We hypothesized that interfering with hnRNPA2B1–RNA interactions at the intracellular level could represent a tool to modulate the homeostasis of several transcripts and their release through EVs, possibly impacting intercellular communication. Taking advantage of a recombinant full-length hnRNPA2B1 protein and previous biochemical characterization of the binding performance to specific RNA sequences [25, 26], we performed a pharmacological screening to find inhibitors of the interaction with a synthetic purine-rich RNA probe. We found six hit compounds that efficiently inhibited the interaction *in vitro*, with some passing a validation journey reaching the interference of EV-RNA quality. By exploiting miR-221 as an elective readout, we observed that hematein and phenotrin could interfere with the vesicular enrichment of this transcript irrespective of the secreted EV abundance. Correlative experiments indicated that the drug counteracted the hnRNPA2B1-induced NF-kB activation in EV recipient cells, demonstrating that EV-RNA quality can be modulated by interfering with post-transcriptional control and exploited to develop therapeutic strategies targeting paracrine functions.

## Materials and methods

### Cell cultures and reagents

Mouse motor neuron-like hybrid cell line (NSC34), human embryo kidney HEK293T, and prostate cancer DU-145 cells were maintained using Dulbecco’s modified Eagle’s medium (DMEM), supplemented with 10% fetal bovine serum, 1% L-glutamine, and 1% penicillin/streptomycin (Life Technologies, Carlsbad, CA, USA) in standard conditions at 37°C with 5% CO_2_.

Induced pluripotent stem cells (iPSCs)-derived small molecule neuroprogenitor cells (smNPCs), produced as described in [27, 28], were differentiated to reach 2-weeks matured motor neurons. The iPSC generation and respective characterizations was previously reported [27] and approved by the local ethics committee (EK45022009). Briefly, smNPCs were seeded on Matrigel (Corning 354 234) coated 12-well plates and maintained in N2/SM1 base medium (made of 48.75% DMEM/F12, 48.75% neurobasal, 1% pen/strep/glut, 1% SM1, and 0.5% N2) supplemented with 3 μM CHIR99021, 150 μM ascorbic acid, and 0.5 μM purmorphamine (PMA). For the differentiation, smNPCs were plated on Matrigel coated six-well-plates and fed with N2/SM1 base medium supplemented with 1 μM PMA, 1 ng/ml BDNF (Brain-derived neurotrophic factor), 0.2 mM ascorbic acid, 1 μM Retinoic acid, and 1 ng/ml GDNF (Glial cell line-derived neurotrophic factor) for 10 days. Then the cells were plated on 15% Poly-L-Ornithine (Merck/Sigma A-004-C) and 1:100 Laminin- (Biotechne 3400-010-02) coated plates and maintained in N2/SM1 base medium supplemented with 5 ng/ml Activin A (for the first day only), 0.1 mM dBcAMP, 2 ng/ml BDNF, 0.2 mM ascorbic acid, 1 ng/ml TGFβ-3, and 2 ng/ml GDNF for two weeks. Then, smNPC-derived motor neurons were exposed to 5 μM of compounds for 6 h.

Primary cortical neurons were cultured from embryonic E15.5 C57BL/6J mice as previously described [29]. TDP-43^Q331K^ mice were conducted as already described [30] according to institutional guidelines, national (D.L. no. 116, G.U. suppl. 40, 18 February 1992, n. 8, G.U., 14 July 1994), and international laws and policies (EEC Council Directive 86/609, OJ L 358, 12 December1987; National Institutes of Health Guide for the Care and Use of Laboratory Animals, US National Research Council, 1996).

### hnRNPA2B1 over-expression and silencing

NSC34 and HEK293T cells were transfected with a plasmid coding for the hnRNPA2B1 (NM_002 137) Human Tagged ORF Clone (Origene, RC219318) using Lipofectamine™ 3000 Transfection Reagent (Invitrogen L3000001). After 48 h of transfection, cells were used for the subsequent analysis. Protein silencing was obtained using small interfering RNAs (siRNAs) targeting *Hnrnpa2b1* or *hnRNPA2B1* genes (ON-TARGETplus mouse Hnrnpa2b1 siRNA-L-040194-01-0005; ON-TARGETplus human HNRNPA2B1 siRNA- L-011690-01-0005). Scramble (SCR) siRNA (ON-TARGETplus Non-targeting siRNA #3, 5 nmol Catalog ID: D-001810-03-05) was used as a control. INTERFERin^®^ (Polyplus, 101000036) was used as a transfection reagent in this case.

### EV isolation and characterization

EVs were isolated from HEK293T, NSC34, and smNPC-derived motor neurons using nickel-based isolation (NBI) or differential ultracentrifugation (UC). NBI protocol was performed as already described [31]. Briefly, after a first centrifugation step at 2800 × *g* for 10 min, nickel beads were incubated for 30 min with serum-free media collected from cells in a ratio of 25 μl of beads per ml of medium. After a 2 min centrifugation at 600 *g*, particles were eluted from the beads by adding 1× elution buffer made of Solution A [16 mM ethylenediaminetetraacetic acid (EDTA), UltraPure, pH 8.0, Thermo Fisher] and Solution B (10 mM NaCl, 225 μM citric acid, Sigma–Aldrich) diluted five times in phosphate-buffered saline (PBS). After 15 min incubation at 28°C in a thermoshaker, the EVs were retrieved by 1-min centrifugation at 1800 × *g* and subjected to the following characterization. NBI was used to recover polydisperse EVs and include the RNA contribution from these for reverse proteomics experiments. The EV-RNA for reverse proteomics was pooled from three consecutive EV isolation procedures performed on a total of 30 T75 flasks (10 flasks each) of NSC34 cells grown until 80% confluence under standard conditions before washing with PBS and incubating with serum-free medium with glutamine and antibiotics. Differential UC was applied as an alternative EV isolation protocol to confirm the detection of hnRNPA2B1 protein. UC was performed by a SW 32 Ti swinging bucket rotor in an Optima XPN-100 ultracentrifuge (Beckman Coulter, Brea, CA, USA). The cell-conditioned serum-free media, after the first centrifugation step at 2800 × *g* for 10 min, was put in open-top Ultra-Clear centrifuge tubes (Beckman Coulter-344058) and subjected to 100 000 × *g* (100 K) UC for 70 min at 4°C under vacuum, with or without an additional step at 10 000 × *g* (10 K) to first sediment larger particles. Then the supernatant was removed, and the pellet was resuspended in 0.22 μm filtered PBS. Differential UC alone, consisting of 2800 × *g* for 10 min and 100 000 × *g* for 70 min, was applied for experiments reported in Fig. 5. The retrieved particles were then used for the subsequent experiments.

EVs from DU-145 cells were isolated using the exoEASY kit (Qiagen, #76064), following the manufacturer’s instructions, as an alternative EV isolation method to confirm the detection of studied miRNAs cargoes.

To characterize recovered particles, nanoparticle tracking analysis (NTA) was applied using NanoSight NS300 instrument (Malvern Panalytical Ltd., Malvern, UK) equipped with a 488 nm blue laser and a sCMOS camera. EV samples were measured in scatter mode and diluted in PBS to have at least 20 particles/frame. Three individual consecutive 60 s videos were acquired for all the EV samples, with camera level 14. Information about particles concentration, mean and mode diameter were retrieved using built-in NanoSight Software NTA3.3.301 (Malvern). A fraction of EV samples recovered for pull-down (PD) experiments was also characterized in fluorescent mode after staining with Cell-Mask Orange (CMO), a dye with affinity for lipids. The quality of recovered particles was also verified by transmission electron microscopy (TEM) and immunoblotting. For TEM experiments, NBI-isolated EVs were sedimented by UC at 100 000 × *g* for 70 min at 4°C to obtain concentrated particle pellets. Five μl of sample were fixed in 2.5% formaldehyde and applied to 300-square mesh copper-nickel grids coated with carbon. The grids were negatively stained with 1% buffered uranyl acetate, pH 4.5, and observed using a TEM FEI Tecnai G2 Spirit microscope operating at 100 kV and equipped with an Olympus Morada side-mount 2K × 4K charge-coupled device camera (magnification used: 20 500×). In immunoblotting experiments, anti-Calnexin (AB22595 Abcam) and GM130 (AB52649 Abcam) antibodies we used for negative markers, while anti-SYNTENIN (AB133267 Abcam) antibody was used for a positive marker.

### PD assay

To prepare native protein lysates, 1–3 × 10^6^ NSC34 cells were lysed in buffer R-Lysis [25 mM HEPES, pH 7.5, 100 mM NaCl, 1× protease inhibitor (Thermo Fisher, 78429)] and subjected to a water-bath sonication (35–40 amplitude, 6–7 cycles of 7 s on and 45 s off). After a centrifugation at 14 000 rpm for 20 min at 4°C, the supernatant was collected and 200 μg were incubated with 5 μl of buffer equilibrated Dynabeads^TM^ M-280 Streptavidin (Invitrogen, 11205D) for 15 min at 4°C in rotation to perform a pre-clearing step. After the magnetic separation, the pre-cleared lysate was incubated with 10 μl of 100 μM RNA biotinylated probes (or 400 ng of biotinylated EV-RNA) and incubated for 1 h at 4°C in rotation. Then, 5 μl/sample of beads were added and the sample were put again in rotation for 20 min at 4°C. After the magnetic separation the beads were washed with buffer R-Lysis (or 100 mM ammonium bicarbonate for proteomics) and bound proteins were eluted with 20 μl of 1× Laemmli sample buffer heating the samples at 95°C for 5 min, otherwise beads were resuspended in 40 μl with 100 mM ammonium bicarbonate for liquid chromatography-mass spectrometry (LC-MS) analysis.

### LC-MS analysis

Proteins bound to the poly-adenylated EV-RNA, hybridized with biotinylated oligo(dT), then captured by magnetic Streptavidin beads (Dynabeads^TM^ M-280 Streptavidin (Invitrogen, 11205D) were subjected to on-bead trypsin digestion. Briefly, samples were reduced and alkylated with dithiothreitol (DTT) 10 mM at 56°C for 45 min and iodoacetamide 20 mM at room temperature (RT) for 30 min in the dark, respectively. One microgram of trypsin (Thermo Fisher Scientific) was added to each sample and the beads were incubated at 37°C overnight with gentle shaking. Following digestion, beads were collected and the supernatant was transferred to a fresh Eppendorf tube. Beads were washed with 50 μl of 100 mM ammonium bicarbonate and the supernatants were pooled. Digested peptides were then acidified with 1% Trifluoroacetic acid (TFA) to a pH 2.5, desalted on C18 stage-tips and resuspended in 20 μl of 0.1% formic acid buffer for LC-MS/MS analysis. Digested samples were separated using an Easy-nLC 1200 system (Thermo Scientific). A 28 cm reversed-phase column (inner diameter 75 μm packed in-house with ReproSil-Pur C18-AQ material: 3 μm particle size, Dr Maisch, GmbH), heated at 40°C, was used for separating the peptides, with a two-component mobile phase system of 0.1% formic acid in water (buffer A) and 0.1% formic acid in 80% acetonitrile (buffer B). Peptides were eluted using a gradient of 5%–25% over 57 min, followed by 25%–40% over 13 min and 40%–98% over 10 min, and kept at 98% over 10 min, a flow rate of 400 nl/min. Samples were injected in an Orbitrap Fusion Tribrid mass spectrometer (Thermo Scientific, San Jose, CA, USA) and data acquired in data-dependent mode (2100 V). Temperature of the ion transfer tube was set at 275°C. Full scans were performed in the Orbitrap at 120.000 full width at half maximum (FWHM) resolving power (at 200 m/z), 50 ms maximum injection time, and an AGC target of 1 × 10e6. A mass range of 350–1100 m/z was surveyed for precursors, with first mass set at 140 m/z for fragments. Each full scan was followed by a set of MS/MS scans (HCD, collision energy of 30%) over 3 s cycle time at 150 ms maximum injection time (ion trap) and AGC target of 5 × 10e3. A dynamic exclusion filter was set every 30 s. Data were acquired using the Thermo software Xcalibur (version 4.3) and Tune (version 3.3). QCloud was used to control instrument longitudinal performance [32].

Peptides searches were performed in Proteome Discoverer 2.2 software (Thermo Scientific) against the Mus musculus FASTA file (uniprot, downloaded April 2021) and a database containing major common contaminants. Proteins were identified using the MASCOT search engine, with a mass tolerance of 10 ppm for precursors and 0.6 Da for products. Trypsin was chosen as the enzyme with five missed cleavages. Static modification of carbamidomethyl (C) and variable modification of oxidation (M) and acetyl (protein N-term) were incorporated in the search. False discovery rate was filtered for < 0.01 at PSM, at peptide and protein levels. Results were filtered to exclude potential contaminants and proteins with less than two peptides. Protein–protein network analyses were generated by STRING 11.5 tool (http://string-db.org) using medium confidence.

### Flow cytometry and dot blot with short RNA probes

NSC34 cells were seeded in six-well plates and transfected by INTERFERin – siRNA/miRNA transfection reagent (Polyplus), following manufacturer’s instructions, with 150 nM of sense (CY3-5′-UAGGGA) and antisense (CY3-5′-AUCCCU) RNA oligos at 70%–80% confluence. At 24 h post-transfection, media were subjected to EV isolation and cells were recovered by simple pipetting with 300 μl/well of PBS. Resuspended cells were loaded on a BD FACSymphony™ flow cytometer and the specific probe signals were recorded in PE-A channel after considering SSC-A/SSC-W sub-gating for excluding cell doublets. Data were reported as a percentage of PE-A-positive events.

Flow cytometry of a fraction of RNA immunoprecipitation (RIP) experiments, consisting of RNA/protein-antibody-bead complexes, was performed on the same instrument using a sequential gating with Streptavidin beads alone ± antibodies (this signal corresponded to background). Pyronin Y (PY) was used at a final concentration of 50 ng/ml and the PY-positive events recorded for 1 min.

Dot blots were performed using media aliquots or EV samples treated or not with 1 μg/ml of RNAse A for 15 min at RT. Briefly, 1–5 μl of samples were spotted on wells of a dot blot apparatus (Bio-Rad) over an Amersham Hybond-N^+^ membrane. Samples were air-dried for at least 1 h at RT under a biological hood. Fluorescent signals were detected on a Typhoon instrument (GE Healthcare).

### Recombinant protein expression and purification

pGEX-6P-1-HNRNPA2B1 plasmid was used to transform BL21 (DE3) competent cells. A pre-inoculum of transformed BL21 (DE3) bacteria cells was grown in Luria Broth (LB) broth (Sigma–Aldrich) supplemented with ampicillin (50 μg/μl) ON at 37°C at 220 rpm. The day after, bacteria were diluted in 1 l LB supplemented with 50 μg/μl Ampicillin and cultured at 37°C at 220 rpm. When optical density at 600 nm reached 0.7, the protein expression was induced with 0.2 mM Isopropyl β-d-1-thiogalactopyranoside (IPTG) for 13 h at 25°C. The culture was harvested by centrifuging at 6371 × *g* for 15 min at 4°C and the pellet resuspended in 30 ml of lysis buffer [50 mM Tris–HCl, pH 7.5, 2 mM EDTA, 200 mM NaCl, 1:1000 lysozyme (stock 25 mg/ml), 1 mM DTT, bacteria protease inhibitor cocktail] and incubated on ice for 20 min. Upon adding 0,1% tween, the lysate was incubated for an additional 20 min on ice followed by sonication (7–8 cycles – 20 s ON, 30 s OFF). Sonicated samples were centrifuged at 13 000 × *g* for 45 min at 4°C. Then the supernatant was filtered (0.45 μm) and incubated with buffer equilibrated Pierce™ glutathione agarose beads (Pierce, 16101) for 2 h at 4°C in rotation. The lysate was loaded in a column and, after the collection of the flow-through (FT), beads were washed with 25 ml of high-salt wash buffer (50 mM Tris–HCl, pH 7.5, 500 mM NaCl). GST-hnRNPA2B1 was eluted in 15 ml elution buffer (100 mM NaCl, 50 mM Tris–HCl, pH 7.5, 0.1 mM DTT, 300 mM glutathione) and concentrated using Amicon Ultra-15 at 4000 × *g* (Merck Millipore).

### AlphaScreen and pharmacological screening

AlphaScreen assay was used to study the interaction between hnRNA2B1 and different biotinylated single-stranded RNA probes: EXO RNA (5′-BTeg- GGGGAGGUUAGGGAGGAGGGGGGUAGGCGCC), RNA 114 (5′- BTeg-AAGGACUAGC), and RNA 276 (5′-B-Teg- AGGACUGC). The assays were performed in OptiPlate-384-well plates (PerkinElmer, 6007299) in 20 μl final volume. The ligands were diluted in AlphaBuffer [25 mM HEPES, pH 7.4, 100 mM NaCl, 0.01% bovine serum albumin (BSA)] and tested using AlphaScreen GST Detection Kit (PerkinElmer, 6760603C). For the assay optimization, different concentrations of the RNA probes (0–100 nM) were incubated with different concentrations of GST-hnRNPA2B1 (0–60 nM) in the presence of streptavidin Donor beads and anti-GST-Acceptor beads (PerkinElmer) (20 μg/ml final concentration). For the reactions, 4 μl of the RNA probes were first added to the plate, then 16 μl of a mix containing all the other components was added, and the plate was incubated at room temperature for 1 h in the dark.

The high-throughput drug screening was performed in a total volume of 20 μl using 17 nM of RNA EXO motif (chosen below the hooking point), 30 nM GST-hnRNPA2B1, and 250 nM of compounds belonging to mass spectrometry (MS) Spectrum Collection library (MicroSource, 2000 compounds). Compounds were dispensed into white 384-well Optiplates (PerkinElmer) using Echo 650 acoustic dispenser (Beckman Coulter) followed by the addition of protein in Alpha Buffer using the EL460 dispenser (BioTek). After 15 min of incubation, RNA probes were added by Echo 650 acoustic dispenser, while Donor and Acceptor beads from the GST detection kit (Perkin Elmer) were dispensed by EL460 instrument. Following 60 min of incubation, Alpha signal was detected using Enspire plate reader instrument (PerkinElmer). The raw Alpha counts were normalized to Dimethyl sulfoxide (DMSO)-treated controls within every plate.

### RNA electrophoretic mobility shift assay

hnRNPA2B1 (110 nM) was incubated with compounds (1 μM) in RNA electrophoretic mobility shift assay (REMSA) Buffer (20 mM HEPES, pH 7.5, 50 mM KCl, 0.5 μg BSA, 0.25% glycerol) at room temperature for 10 min. Then, 30 nM RNA EXO were added in a final volume of 20 μl, and the reaction mix incubated at room temperature for 50 min. The mix was then loaded in a 4% native polyacrylamide gel containing 2% glycerol and run in 0.5× Tris Borate EDTA (TBE) buffer at 60 V for the first 15 min and at 80 V for additional 60 min. The RNA probe signal was detected using Typhoon Instrument (Amersham™ Typhoon™ 5-29187191) using filters for infrared emission detection.

### RNA immunoprecipitation

Approximately 2 × 10^6^ cells were used for each RIP experiment, performed as already described [33], without cross-linking steps and using 0.5 μg/ml of antibodies. Beads-precipitated samples were divided for immunoblotting or TRIzol-based RNA isolation. Densitometric analysis obtained using Image J software (v.1.54). miRNA TaqMan probes were bought from Thermo Fisher with the following codes: miR-221 (477 981), miR-1910 (479 581), and miR-126 (4 427 975).

### RNA extraction and cDNA synthesis

RNA from cells was extracted using TRI Reagent^®^ (T9424, Sigma) following the manufacturer’s instructions. Thermo Fisher Nanodrop 2000 Spectrophotometer was used to assess the purity and quantity of extracted RNA. RNA from EVs was extracted using Single Cell RNA Isolation Kit (51800, Norgen) following the manufacturer’s protocol. The elution was in 10–20 μl of RNase-free water and the RNA profiling and quantification was assessed by Agilent 2100 Bioanalyzer and RNA 6000 Pico Kit (5067-1513). Cell- and EV-RNAs were also analysed with DNF-472 HS RNA (15 nt) Kit (Agilent Technologies, Santa Clara, CA), according to the manufacturer. Briefly, 2 μl of RNA samples concentrated within a range of 50–5000 pg/μl and RNA Ladder were added to Diluent Marker, denatured at 70°C for 2 min and loaded. Results were analysed with Agilent ProSize data analysis software, that allows the analysis of RNA size, concentration and quality. Through a Peak Analysis it was possible to access the concentration of each peak recognized by the software. Half a nanogram of extracted RNA from cells and EVs was used as input for complementary DNA (cDNA) synthesis. cDNA was produced using TaqMan™ Advanced miRNA cDNA Synthesis Kit (Applied Biosystems™, A28007) following manufacturers’ instructions and used for droplet digital polymerase chain reaction (PCR) experiments.

### Droplet digital PCR

For hsa-miR-221–3p detection in EVs and in cells, 1:200 cDNA was mixed with 1× hsa-miR-221–3p Advanced miRNA Assay (477 981_mir, Thermo Fisher – A25576) and ddPCR Supermix for Probes (Bio-Rad – 1863026) to a final volume of 23 μl. Y3 RNA was used as a reference. The same diluted cDNA was mixed with 50 nM Y3 primers (mouse: mY3 Fw: 5′-GGTTGGTCCGAGAGTAGTGG-3′, mY3 Rv: 5′-AAAGGCTGGTCAAGTGAAGC-3′; human: hY3 Fw: GGCTGGTCCGAGTGCAGTG, hY3 Rv: GAAGCAGTGGGAGTGGAGAA) and QX200 ddPCR EvaGreen Supermix (Bio-Rad, 1864033) to a final volume of 23 μl. Droplet formation and PCR conditions were performed following the manufacturers’ instructions using ddPCR™ 96-well plate (Bio-Rad). QX200 Droplet Reader Bio-Rad was used to read the plates. Analysis and target quantification were performed using QuantaSoft^TM^ Analysis Pro Software.

### Immunoblotting

Cells were lysed in lysis buffer [50 mM Tris–HCl, pH 7.4, 150 mM NaCl, 1 mM EDTA, 0.25% NP-40, 0.1% Triton X-100, 0.1% sodium dodecyl sulphate, 1× protease inhibitor (Thermo Fisher, 78429)]. Cell lysates were loaded on 10% acrylamide gels and transferred to a polyvinylidene difluoride (PVDF) membrane. Membranes were incubated with 1:1000 primary antibody and 1:10 000 peroxidase-conjugated secondary antibodies in 3% milk in Tris-buffered saline with Tween-20 (TBST). The signal was measured with Amersham ECL HRP-Conjugated Antibodies (Cytiva) using BioRad Chemidoc XRS+.

The following primary antibodies were used: anti-hnRNPA2B1 (PA5-34939, Invitrogen), anti-Calnexin (Abcam), anti-MycTag (Proteintech), anti-NF-kB-p65 (ab76311) The following secondary antibodies were used: goat anti-rabbit (Jackson ImmunoResearch Laboratories, Inc.), goat anti-mouse (Jackson ImmunoResearch Laboratories, Inc.). Band areas and pixel intensities were quantified using ImageJ software.

### Absorbance scan and fluorescence indicator displacement

Five μM RNA EXO motif were mixed with 25 μM compounds in AlphaBuffer (25 mM HEPES, pH 7.4, 100 mM NaCl, 0.01% BSA) to a final volume of 20 μl. Absorbance spectrum (200–530 nm) was measured using Tecan Spark^®^ microplate reader with a NanoQuant plate at room temperature. In fluorescence indicator displacement (FID), a 2× final concentration of Midori green advanced (Resnova) was added to the samples and the melting curve measured using Bio-Rad CFX96™ System from 30°C to 90°C.

### Immunofluorescence staining

Cells were washed twice with PBS without Ca^2+^/Mg^2+^ (LifeTechnologies) and fixed with 4% Paraformaldehyde (PFA) in PBS for 10 min at room temperature, afterwards washed three times with PBS. Fixed cells were permeabilized for 10 min in 0.2% Triton X solution and then incubated for 1 h at RT in blocking solution (1% BSA, 5% donkey serum, 0.3 M glycine and 0.02% Triton X in PBS). Primary antibodies were diluted in blocking solution and cells were incubated with primary antibody solution overnight at 4°C. The following primary antibodies were used: rabbit anti-Islet (1:500, Abcam), goat anti-ChAt (1:500, Millipore), mouse anti-MAP2 (1:500, BD Biosciences). Nuclei were counter stained using Hoechst (LifeTechnologies).

### Statistical analysis

MS downstream analysis were performed using the ProTN proteomics pipeline (www.github.com/TebaldiLab/ProTN and www.rdds.it/ProTN). Briefly, peptide intensities were log2 transformed, normalized (median normalization) and summarised into proteins (median sweeping) with functions in the DEqMS Bioconductor package [34]. Imputation of the missing intensities was executed by PhosR package [35]. Differential analysis was performed with the DEqMS package, proteins with *P*-value < .05 were considered significant. Functional enrichment analysis of differentially expressed proteins was performed with EnrichR [36]. Enriched terms with *P*-value < .05 and overlap size > 4 were considered significant.

Additional statistical analyses were performed using *t*-test in the GraphPad Prism^®^ software, version 9.0. Comparisons were considered statistically significant if *P*-values were < .05 (*), < .01 (**), or < .001 (***).

## Results

### Reverse proteomics reveals a competition of hnRNPs for secreted vesicular RNA

**Figure 1.**
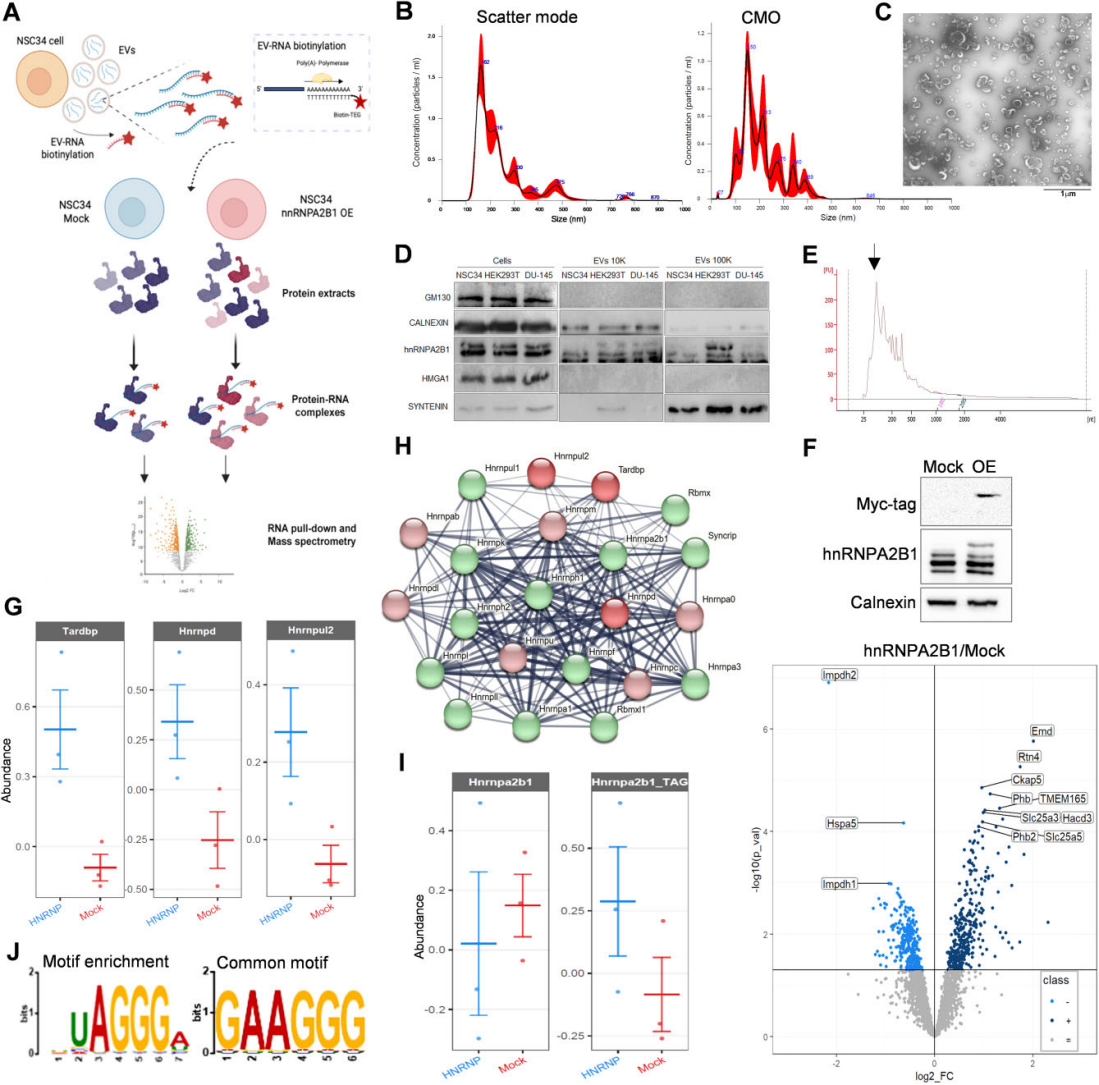
Reverse proteomics using the EV-RNA to prioritize RBP binding partners. (**A**) Experimental workflow of reverse proteomic approach. The RNA was extracted from EVs and then enzymatically polyadenylated. A biotinylated oligo(dT) was hybridized and then incubated with magnetic Streptavidin beads to constitute the heterogeneous protein-capturing probe. Streptavidin beads with biotinylated oligo(dT) alone were included as technical negative controls. The probe was then incubated with cell lysates overexpressing or not hnRNPA2B1. Precipitated proteins were analysed by MS/MS and reported as a ratio between the two different cell lysates and from three independent experiments. The image was created on biorender.com. (**B**) Representative NTA profile of NSC34-recovered particles detected in scatter mode (left) and corresponding lipid particles positive to CMO detected in fluorescence mode (right). (**C**) Representative TEM negative stain acquisition of EVs recovered from NSC34 cells by NBI and then ultracentrifuged at 100 000 × *g* for 70 min at 4°C. A 20 500× magnification is shown. The scale bar corresponding to the original one embedded in gray, was added below for better recognition. (**D**) Immunoblotting using lysates of NSC34, HEK293T, and DU-145 cells and released EVs. Anti-GM130 and CALNEXIN primary antibodies were used as negative EV markers, while anti SYNTENIN antibody was used as positive EV marker. The gel is one representative of three independent experiments. (**E**) Representative bioanalyzer profile of the EV-RNA subjected to polyadenylation for reverse proteomics experiments. EV-RNAs were pooled as described in the experimental section. The black arrow indicates the enrichment of RNA fragments between 100 and 200 nt expected from vesicular RNAs. (**F**) Immunoblotting on NSC34 lysates expressing (OE) or not (Mock) the Myc-His-hnRNPA2B1 protein, recognized by anti-hnRNPA2B1 or anti-Myc primary antibodies. The shown gel is a representative of several independent experiments. Below, the volcano plot shows the differentially enriched or depleted proteins detected by MS and reported as a ratio between hnRNPA2B1 OE and Mock lysates. Enriched proteins (Class “+”), depleted proteins (Class “−”), and unchanged proteins (Class “=”) are indicated. (**G**) Relative abundance, in three independent experiments, of selected hnRNPs significantly enriched in the hnRNPA2B1 OE condition. (**H**) Network of experimentally validated interactions among the identified hnRNPs obtained from STRING database (https://string-db.org). Red circles indicate hnRNPs significantly enriched in the ratio OE/Mock; pink circles indicate hnRNPs showing a trend of enrichment; Green circles indicate hnRNPs showing a trend of displacement. Raw data are available in Supplementary Table S1. (**I**) Relative abundance of recombinant (Myc-His-hnRNPA2B1) and endogenous (hnrnpa2b1) proteins in the ratio OE/Mock. (**J**) Sequence motifs analysed by MEME toolbox (https://meme-suite.org/meme) using experimentally validated RNA sequences present in RPDB database (http://rbpdb.ccbr.utoronto.ca) and recognized by the identified hnRNPs..

Several RBPs, such as hnRNPA2B1, were circumstantially implicated in vesicular RNA (EV-RNA) sorting, given the recognition of specific consensus sequences found in vesicular transcripts [6, 22, 23]. To prioritize RBP–RNA interactions that could significantly impact the EV-RNA quality, we designed a reverse proteomic strategy [37] using the heterogeneous EV-RNA fragments with “retrospective” pulls of proteins (present at the intracellular level) to PD protein binders *in vitro*. By this approach, we assumed that available RBPs could competitively recognize eligible RNAs according to transcript quality. We optimized the capture probe by hybridizing EV-RNA fragments with a 5′-biotinylated poly(T) oligo, subsequently incubated with streptavidin beads to PD protein–RNA complexes. We first identified enriched proteins over oligo-streptavidin beads alone. Then, to normalize the relative protein abundance in cell lysates and get insights on competition, we applied the same EV-RNA probe in parallel with lysates from cells expressing or not a recombinant hnRNPA2B1 protein. MS was then applied to identify binding partners, also reported as a ratio between the two cell lysates (Fig. 1A).

To obtain the EV-RNA probe composed of heterogenous fragments, we recovered EVs released from NSC34 cells by NBI [31, 38] and profiled them by NTA. As previously reported [38], we observed polydisperse particles ranging from 100 to 800 nm, with small population peaks comprised between 160 and 220 nm (Fig. 1B, scatter mode). To assess the presence of lipid particles, we also profiled samples in fluorescent mode after staining with CMO, a dye for lipid membranes. We detected about 75% of CMO-positive particles, confirming the presence of EVs with expected sizes in our preparations (Fig. 1B, CMO). The quality of recovered particles was analysed by TEM, showing size-heterogeneous vesicles with an almost round-shape morphology (Fig. 1C). The common protein marker profiles of these EVs were obtained by immunoblotting after combining NBI with differential UC, allowing the sediment of larger particles at 10 000 × *g* (10 K) and smaller particles at 100 000 × *g* (100 K), respectively (Fig. 1D). We characterized in this way the EVs secreted by all the cell lines used in the study (NSC34, HEK293T, and DU-145), clearly showing the selective enrichment of SYNTENIN in the smaller EVs, in contrast to CALNEXIN and GM130 negative markers. Notably, we systematically detected hnRNPA2B1 protein isoforms in both 10K and 100K fractions, in contrast to HMGA1, another protein that prevalently localizes into the nucleus. This set of experiments confirmed the validity of the EV isolation methods applied and a ubiquitous hnRNPA2B1 protein cargo of EVs secreted from these cells. We collected EVs from NSC34 cells to prepare the EV-RNA probe for reverse proteomics. We isolated the RNA from ∼10^12^ pooled particles and profiled it by Bioanalyzer, confirming the presence heterogeneous fragments with a typical peak between 100 and 200 nt [38, 39] (Fig. 1E). Subsequently, 0.5 μg of EV-RNA were enzymatically polyadenylated to obtain complementarity with a 5′-biotinylated poly(T) oligo. We incubated this heterogeneous RNA source with Streptavidin-beads and performed a PD of protein–RNA complexes at equilibrium, without cross-link steps [33], in control (Mock) and hnRNPA2B1-expressing (OE) cell lysates (Fig. 1F, western blotting) for the MS analysis. From the protein ratio, we expected to observe fluctuations reflecting a higher affinity (enriched proteins) or a competition (depleted/displaced proteins) of candidates upon ectopic expression of hnRNPA2B1.

We found 336 significantly enriched proteins and 279 significantly depleted proteins in hnRNPA2B1 versus Mock samples (Fig. 1F, volcano plot, and Supplementary Table S1). The enriched proteins populated the ontologies of RNA-binding, protein transport, and organelle organization with components of membranes and vesicular trafficking (Supplementary Fig. S1A). On the other hand, the depleted proteins mainly described the same ontologies but distinguished proteins involved in the immune response. In the protein-enriched dataset, ∼4% of terms (14 out of 336) belonged to the Rab protein family, which significantly emerged in the first molecular function ontology (Guanosine diphosphate (GDP) binding, *P-*value= 4.97 × 10^−12^) together with other Rab proteins showing a trend of enrichment (*n* = 13) or depletion (*n* = 3) (Supplementary Table S2). The second relevant GO (RNA binding, *P-*value= 2 × 10^−11^) was populated by RBPs (∼18%), with Tardbp, Hnrnpd, and Hnrnpul2 as remarkably enriched (Fig. 1H, red circles). This cluster included other 21 hnRNP interacting members showing a trend of accumulation (Fig. 1H, pink circles) or instead of displacement with respect to the Mock condition (Fig. 1H, green circles). The human hnRNPA2B1 was specifically distinguished, especially with respect to the endogenous hnRNPa2b1 protein, using the Myc-His tags and other regions of the primary sequence. The ectopic protein was enriched in contrast to the endogenous Hnrnpa2b1, which showed a displacement trend compared to Mock (*P-*value= 0.12) (Fig. 1I). These data indicated a selective dynamic on specific protein subsets, including direct and indirect protein–RNA interactions. Consistently, 49 out of 336 up-regulated proteins (∼14.6%) clustered in a dense interaction network with at least nine reported hnRNPA2B1 interactors, including Tardbp and Hnrnpul2 (Supplementary Fig. S1B). Focusing on the quality of the EV-RNA responsible for these protein fluctuations, these interactions could indicate proteins converging on the same transcript, with a competitive displacement of proteins recognizing similar RNA consensus motifs [6, 40]. To better understand this aspect, we first repeated the RNA PD on NSC34 cell lysates upon ectopically expressing a recombinant TDP43, one of the top-ranked hnRNPs (Supplementary Table S1). Remarkably, MS results showed TDP43 among the top binders, together with factors that again populated RNA-binding and vesicular trafficking ontologies, but also specific mitochondrion organization components known to be associated with TDP43 function [41, 42] (Supplementary Fig. S1C and Supplementary Table S3). These data confirmed the validity of reverse proteomics approach and the imposing association dynamics of hnRNPs on the EV-RNA probe *in vitro*. Subsequently, to get insights into substrate preferences of the identified hnRNPs, we searched for experimentally-validated RNA sequences using the identified RBPs as query in the RPDB database [43]. We collected 129 sequences and performed a motif discovery and enrichment analysis using the XSTREME (MEME suite 5.5.1) toolbox. We obtained 20 RNA motifs between 6 and 15 nt in length (Supplementary Fig. S1D), with the UAGGGA motif resulting as the most enriched (*P-*value= 1.78 × 10^−3^) and the GAAGGG motif as the most common among the hnRNPs (*e*value = 3.02 × 10^−1^) (Fig. 1J). Notably, these purine-rich motifs distinguished one of the EXO motifs previously identified in secreted EV-miRNAs and bound by hnRNPA2B1 in T cells [22], as well as motifs we recently identified by a cell-EV transcriptomic analysis orchestrated by hnRNPA2B1–APE1 interaction [44].

Albeit technical limitations potentially associated with sub-optimal oligo-T hybridization, EV-transcripts polyadenylation heterogeneity, and biochemical conditions to test the cumulative affinity of individual proteins, hnRNPs constituted a dynamic network competing for a plethora of transcripts that are *bona fide* secreted as cargo of EVs, with hnRNPA2B1 as a central node of this network.

### Human recombinant hnRNPA2B1 binds to purine-rich ssRNAs

**Figure 2.**
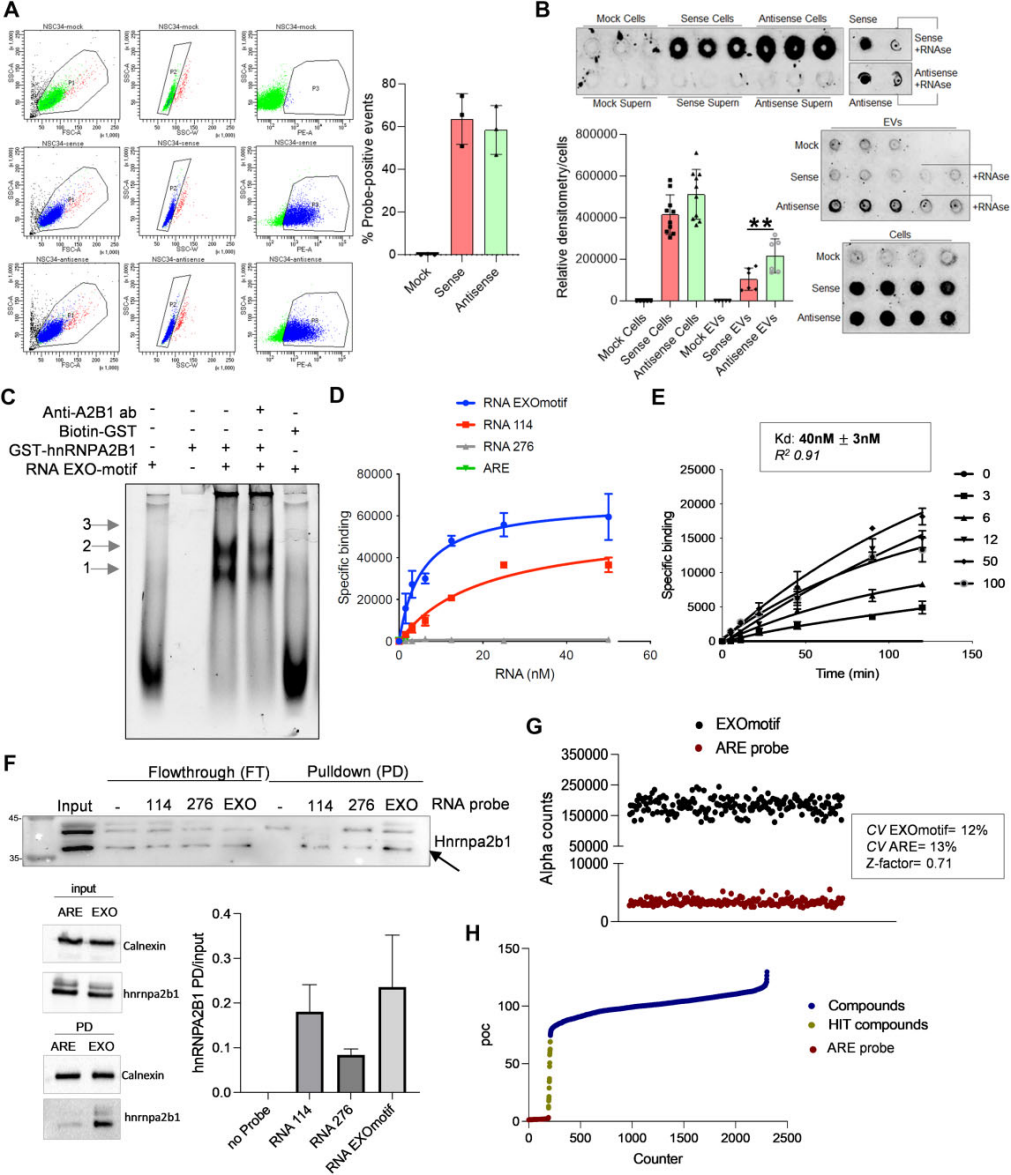
The interaction of recombinant hnRNPA2B1 with purine-rich RNA constitutes a platform for high-throughput drug screening. (**A**) Representative flow cytometry acquisition of NSC34 cells transfected for 24 h with 150 nM with transfection reagent alone (Mock, sense, and antisense fluorescent RNA oligos). Forward and side scatters were chosen to obtain the best cell distribution over the threshold line of the background (population P1). Subgatings in the SSC-A/SSC-W plot were considered to avoid including potential cell doublets, as shown by population P2, before recording the probe signals in the PE-A channel (population P3). Histogram reports mean and data distribution of three independent experiments. (**B**) Representative dot blot performed as described in the experimental section. Treatment with RNAse A was performed with 1 μg/ml of enzyme. Sense and antisense control probes were spotted at 1 nM concentration. Histograms reports data of several experiments; the indicated significance corresponds to a *P*-value < .01. (**C**) Representative REMSA performed at equilibrium with 110 nM of GST-hnRNPA2B1 or Biotin-GST and 25 nM of RNA probe. Arrows indicate protein–RNA complexes with increasing molecular weights and protein oligomerization phases on the same RNA molecules. Supershifts with primary antibody are poorly detectable for the different high molecular weight complexes formed in these conditions. (**D**) Representative plot of saturation binding AlphaScreen experiments to measure the GST-hnRNPA2B1 binding to different biotinylated RNA probes, as described in the text. Dissociation constants (*K*_d_) were determined from nonlinear regression one-site binding model in GraphPad Prism^®^, version 9.0. Mean and standard deviation values derive from two independent experiments with separate GST-hnRNPA2B1 protein purifications. (**E**) Kinetic experiment carried out with 30 nM of GST-hnRNPA2B1 and increasing concentrations of RNA EXOmotif. Association (*K*_on_) and dissociation (*K*_off_) rate constants and equilibrium dissociation constants (*k*_off_*/k*_on_) were determined using nonlinear regression association kinetic model of multiple ligand concentration in GraphPad Prism^®^, version 9.0. Mean and standard deviation derive from two independent experiments with the two protein purifications used in panel (C). (**F**) Representative immunoblotting with anti-hnRNPA2B1 antibody following EXOmotif-, RNA 114-, and RNA 276-based RNA PD. These experiments were paralleled by an immunoblot showing the higher affinity of the EXOmotif RNA probe compared to the ARE probe. Densitometric quantification of protein fractions were normalized to the input. Mean and SD derive from three independent experiments. (**G**) Distribution of positive (EXOmotif) and negative (ARE) protein–RNA probe interactions calculated in the primary drug screening. Relative coefficient of variation (CV) and Z-factor value are indicated. (**H**) Plot of compounds ranked according to percent of control (POC) normalized (DMSO) values.

To verify by orthogonal approaches whether the GAAGGG motif is enriched into secreted EV-RNA fragments, we designed two short, fluorescent RNA oligos, the first one complementary to the motif (CY3-AUCCCU, named “antisense”) and the second one corresponding to a complement control probe (CY3-UAGGGA, named “sense”). After confirming the hybridization to cell- and EV-RNA *in vitro* at room temperature, we transfected NSC34 cells with these oligos to assess their distribution at intracellular and secreted EV levels. The specific signals of the two probes were remarkably detected by conventional flow cytometry 24 h post-transfection, with no statistically significant differences between the two probes in staining more than 50% of cells (Fig. 2A). EVs recovered from the media of these cells indicated a significant accumulation of the antisense probe revealed by dot blots, excluding signals in the same supernatants after EV sedimentation and confirming the sensitivity to RNAseA degradation (Fig. 2B). Altogether, these results consistently indicated the emerged motif as a preferential portion of the EV-RNA cargo.

To explore the purine-rich RNA as a binding substrate and verify the formation of protein–RNA complexes *in vitro*, we designed a corresponding single-stranded RNA oligo (5′-GGGGAGGUUAGGGAGGAGGGGGGUAGGCGCC, or EXOmotif, Supplementary Fig. S2A). We took advantage of previous literature to characterize the RNA-binding activity of hnRNPA2B1 [25] and purified a human full-length, GST-tagged protein expressed in *Escherichia coli* cells. We obtained eluates of ∼2 μM concentration as compared to Coomassie-stained bovine serum albumin standards in the gel. The full-length protein in the working eluate constituted nearly 90% of all detected proteins as compared to other fractions obtained during the purification procedure (Supplementary Fig. S2B and C)

The recombinant protein was used in RNA electrophoretic mobility shift assays (REMSAs) together with a 5′-labeled infrared dye oligo. The artificial RNA ligand generated detectable protein–RNA complexes and protein oligomerization (see arrows in Fig. 2C), independently from the GST tag and partially super-shifted by an anti-hnRNPA2B1 antibody. Next, to gain more quantitative and specificity data on this interaction, we set up AlphaScreen assays with a biotin-labeled version of the synthetic RNA, including two shorter RNA oligos (RNA 114: 5′-AAGGACUAGC and RNA 276: 5′-AGGACUGC) already characterized for the affinity with an hnRNPA2B1 isoform [45], and an AU-rich element (ARE) [46] equivalent in size as negative control. The numbers 114 and 276 corresponded to the observed *K*_d_ values on these probes [45], therefore, we expected a better affinity with RNA 114. We determined the best ligands:beads ratio [46] (hooking point, Supplementary Fig. S2D) and then performed saturation binding experiments (Fig. 2D). The interaction was detected in the nanomolar range and showed the highest affinity for the EXOmotif probe (*K*_d_ = 3.4 ± 1.6 nM) compared to the other ligand (RNA 114), since RNA 276 and ARE oligos showed no relevant interaction with near-to-background Alpha counts. Binding kinetic experiments with the EXOmotif probe, in the time frame of 2 h, indicated both association (*k*_on_= 523 664 M^−1^min^−1^) and dissociation (*k*_off_= 0.02 091 min^−1^) rate constants with an observed *K*_d_ (*k*_off_*/k*_on_) of 40 ± 3 nM (Fig. 2E). Since the calculated *K*_d_ values significantly differed (3.4 versus 40 nM) between saturation and kinetic experiments, we ascribed this discrepancy to a protein oligomerization phase impacting the interaction dynamics. Oligomerization of hnRNPA2B1 was already reported [47] and could be appreciated by EMSA (Fig. 2C) and AlphaScreen with a calculated Hill coefficient of 0.3 (Supplementary Fig. S2D). These results strongly supported the purine-rich sequence as a preferential substrate *in vitro* for the protein. Therefore, we further explored the competitive recognition of this RNA probe by RNA PD experiments. We incubated the biotinylated RNA probes with NSC34 native lysates and recovered protein–RNA complexes by streptavidin magnetic beads. The subsequent immunoblotting showed the precipitation of the endogenous Hnrnpa2b1 protein, displaying multiple bands likely corresponding to post-translationally modified isoforms [48], only in the presence of RNA probes. In these assays, all three probes worked with the EXOmotif > RNA 114 > RNA 276 affinity ranking, although a modest variability was observed with the purine-rich sequence (Fig. 2F).

In line with reverse proteomic findings, these data prioritized purine-rich sequences as relevant substrates for, at least, hnRNPA2B1 at the intracellular level. With the idea of acquiring a tool to interfere with post-transcriptional control and perturb the secreted EV-RNA, we used the AlphaScreen platform to conduct a pharmacological screening to find small molecule inhibitors of the protein–RNA interaction.

### Pharmacological screening and orthogonal validation of six drug inhibitors of the interaction with purine-rich RNA

**Figure 3.**
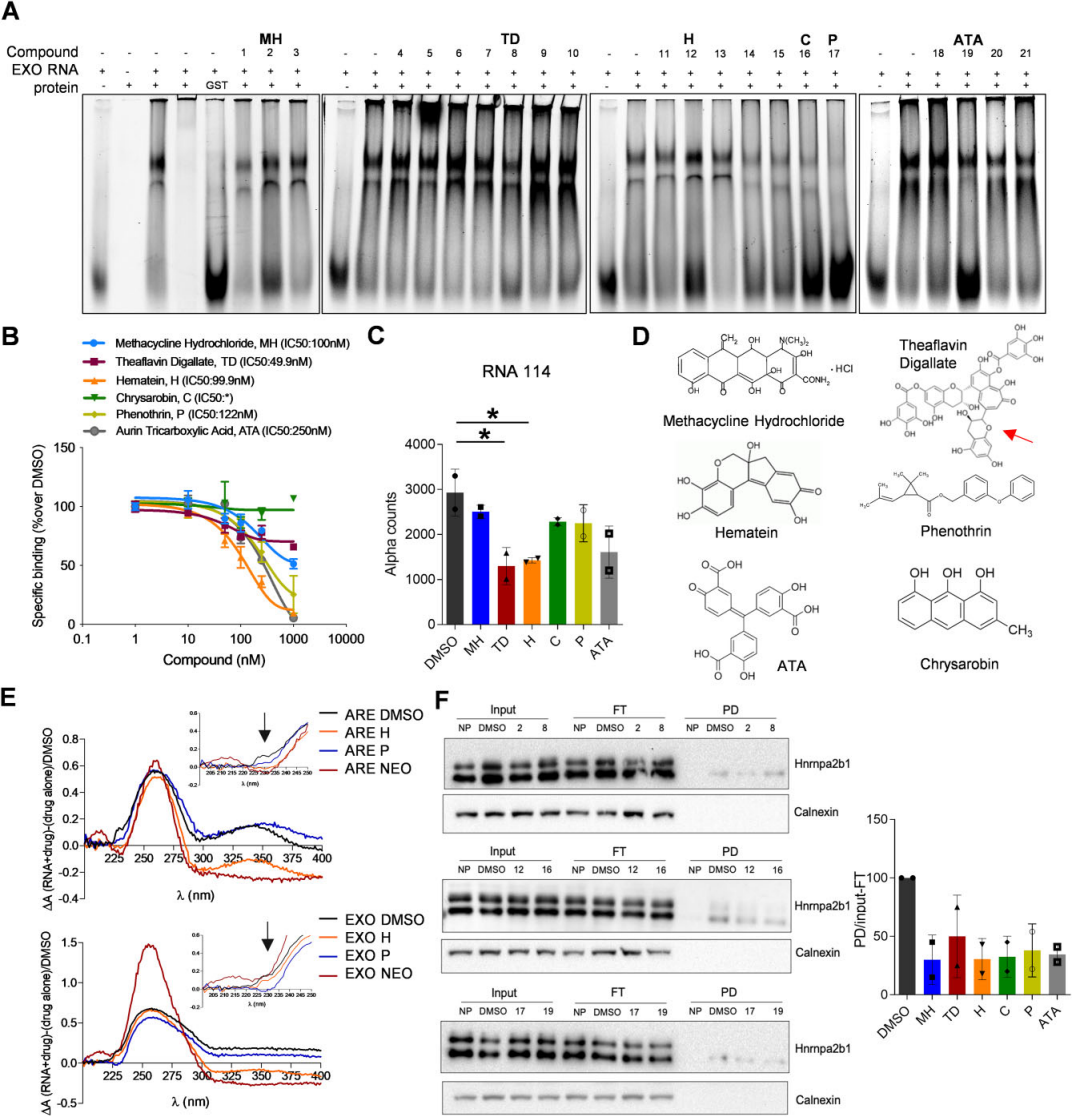
Orthogonal validation of small molecules by biochemical and cell-based assays. (**A**) Counter screening by RNA electrophoresis mobility shift assay (REMSA) showing the activity of AlphaScreen-hit compounds on reducing the hnRNPA2B1–RNA complex formation. (**B**) AlphaScrenn dose-response curves with RNA EXOmotif. The IC_50_ was determined from nonlinear regression using one site fitting model of GraphPad Prism 9. The “*” in the figure indicates “incomplete inhibition”. (**C**) AlphaScreen using 14 nM of RNA 114 probe and 250 nM of compounds. (**D**) Chemical structure of the six AlphaScreen/REMSA hit compounds. Right-hand arrow indicates a chemical space of Theaflavin digallate shared with a hematein. (**E**) Absorbance spectra relative to hematein and phenothrin mixed with RNA EXO motif or ARE probe. Arrows in the inserts magnify the main spectroscopic changes retrieved at 230 nm. (**F**) RNA PD and immunoblotting of Hnrnpa2b1 using the biotinylated RNA EXOmotif probe upon cell treatment with compounds. Densitometric quantification of the protein is reported as fraction normalized on input-FT and DMSO. Histogram reports data of two independent experiments.

We optimized the AlphaScreen assay to obtain the highest signal-to-background ratio and challenge the hnRNPA2B1-EXOmotif interaction with small molecules. We tested a library of almost 2000 compounds plus control DMSO with precautions towards the Alpha assay interference, i.e. using a sub-optimal hooking point and 250 nM of compounds [33]. We calculated a CV of 12% for the positive and 13% for the negative RNAs, and a Z-factor of 0.71 indicating a good performance of the primary screening (Fig. 2G). Compounds were ranked by POC and internal references, such as the biotin interfering with streptavidin Donor beads, were excluded from the preliminary short list of inhibitors, representing 1% of the whole library (Fig. 2H). Counter-screening of 21 hits by REMSAs (Fig. 3A) filtered out six small molecules (methacycline hydrochloride or MH, theaflavin digallate or TD, hematein or H, chrysarobin or C, phenotrin or P, and aurin tricarboxylic Acid or ATA) that were subsequently titrated by alpha assays in dose-response curves (Fig. 3B). H, P, and ATA showed almost complete inhibition of the interaction with IC_50_ of about 100 nM, 120 nM, and 250 nM, respectively. The six hits were also tested at 250 nM against the RNA 114 probe and all of them showed a degree of interference, being H and TD the most effective inhibitors (Fig. 3D). Planarity appeared as a common structural similarity feature among the hit compounds, with an interesting chemical space shared by H and a portion of the TD (red arrow, Fig. 3D).

To have an indication of such ligand interference *in vitro*, we acquired absorbance profiles between 200 and 530 nm at room temperature, testing EXO-motif and ARE probes in a 1:5 micromolar ratio with compounds, including neomycin as a positive control [49, 50]. We observed spectroscopic changes induced by H and P. In particular, H induced absorbance shifts detectable with both RNA probes and resembling those of neomycin in the range of 300–400 nm, instead slightly impacted by P (Fig. 3E). At 230 nm, P induced the main spectroscopic changes with the EXO-motif RNA probe, possibly indicating an affinity for secondary structures [49]. These notions were paralleled by a fluorescence indicator displacement (FID) experiment [50] showing detectable thermal shifts of the EXO-motif RNA of 6.3°C with neomycin, 9.5°C with H, and no detectable shifts with P (Supplementary Fig. S3). In the same settings, none of the compounds caused relevant melting shifts of the ARE-containing RNA probe. These data suggested that at least H and P could recognize purine-rich sequences with a certain degree of affinity, possibly depending on primary sequence and/or secondary structures, impacting downstream protein–RNA interactions. This evidence supported further experiments in a cell-based system. We treated NSC34 cells for 6 h with 5 μM of compound concentration (far from inducing cell death). The short-term treatment was chosen to minimize potential transcriptional effects induced by the drug, therefore preventing a reasonably expected exacerbated RNA unbalance/adaptation to long-term treatments responsible for a decreased cell viability observed at concentration higher than 10 μM. After washing with PBS to remove potential drug residues and cell recovering, we applied the same RNA PD protocol described in Fig. 2D. All the tested compounds prevented the association of hnRNPA2B1 protein with the exogenous purine-rich RNA probe (Fig. 3F), being MH and H the most effective ones. These data substantiated the possibility of interfering at the post-transcriptional level with purine-rich sequence recognition upon cell treatment.

### Small molecules interfered with secretion of hnRNPA2B1-regulated miRNAs

**Figure 4.**
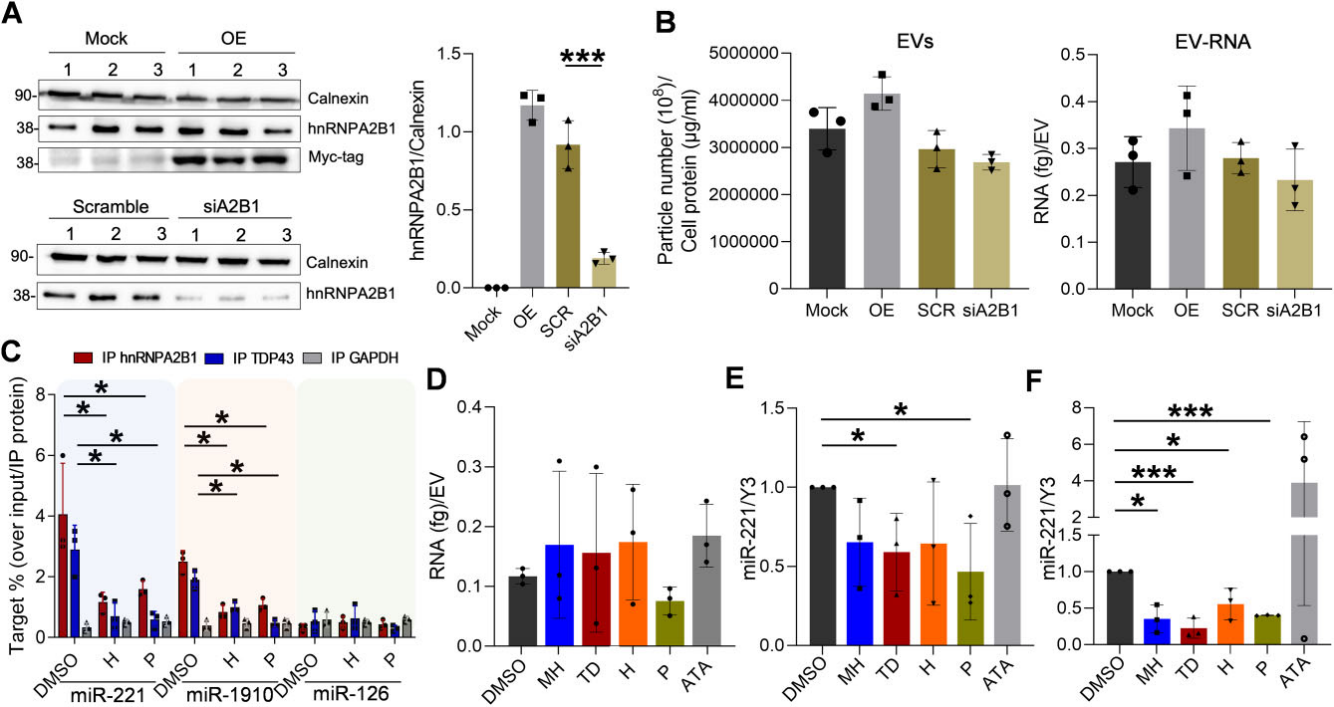
Post-transcriptional activity of small molecules at intracellular and EV-RNA levels. (**A**) Immunoblotting using protein lysates from NSC34 cells transfected with the plasmid encoding the Myc-tagged hnRNPA2B1 (OE) or without DNA (Mock) and with siRNA pools targeting hnrnpa2b1 (siA2B1) or scramble (SCR). Densitometric quantification of all bands was plotted as a ratio with Calnexin. Mock and OE conditions in the histogram refer to the Myc-tag bands. (**B**) Particle concentration determined by NTA using Nanosight NS300 (left). Data were reported after normalization with relative cellular protein content in each condition. The EV-RNA concentration determined by Bioanalyzer was normalized on data reported on the left upon protein expression or silencing (right). Histograms report data of three independent experiments. (**C**) RIP analysis using cytoplasmic lysates of cell treated with compounds and anti-hnRNPA2B1–TDP43, and-GAPDH primary antibodies. IgG-conjugated magnetic beads were recovered and divided for protein detection and RNA isolation. Equivalent amount of IP RNA was used for cDNA synthesis and digital droplet PCR with TaqMan probes for miR-221, miR-1910, and miR-126 detection. The transcript copy number was normalized on relative protein levels and DMSO condition. Histogram reports data of three independent experiments. The indicated significance corresponds to a *P*-value < .05. (**D**) Relative abundance of vesicular RNA recovered after cell treatment and normalized on the particle number detected by NTA. (**E**) Relative miR-221–3p copy number detected by ddPCR from NSC34-derived EV-RNA and cDNA synthesis upon compound treatments. Transcript copy number was normalized on Y3 RNA levels and DMSO condition. (**F**) Relative miR-221–3p copy number detected by ddPCR from motor neurons-derived EVs upon compounds treatment. Transcript copy number was normalized on Y3 RNA levels and DMSO condition. *P-value < .05; *** *P*-value < .001; ****P-value < .0001.

To have an indication of the extent of secreted EV-RNA perturbation, we first evaluated the secretome of cells characterized by an altered dosage of hnRNPA2B1. We transfected NSC34 cells to express a Myc-tagged hnRNPA2B1 protein or silence the endogenous protein by siRNA pools (Fig. 4A). At 48 and 72 h post-transfection, respectively, the recombinant protein was detected by immunoblotting, and there was an 80% reduction of the endogenous counterpart in silenced cells. Corresponding media exposed to those cells were used for EV isolation. The relative concentration of particles detected by NTA, normalized to the relative cellular protein content at the end point, was slightly perturbed with no statistically significant changes compared with Mock or SCR conditions, respectively (Fig. 4B, left). Further, the EV-RNA profiles of the same samples did not show obvious qualitative changes (Supplementary Fig. S4A). However, the EV-RNA abundance appeared highly variable and fluctuating according to protein dosage (Fig. 4B, right). The observed variability could be consistent with a perturbation of selected transcripts, as already demonstrated in hnRNPA2B1-silecing experiments and different cellular models [22, 51, 52]. Therefore, we decided to perform RIP analysis on cytoplasmic lysates to verify if compound treatment can interfere with the recognition of specific RNA targets at the intracellular level. We focused on miR-221 and miR-1910, both harboring a similar purine-rich motif [22] in contrast to miR-126. We treated NSC34 cells for 6 h with DMSO, H, and P (the two most effective compounds). At the end of the treatment, we performed RIP experiments using anti-hnRNPA2B1 and -TDP43 antibodies in parallel with anti-GAPDH as negative control. As shown in Fig. 4C, both hnRNPs significantly enriched miR-221 and miR-1910, in contrast to miR-126 and GAPDH immunocomplexes. Interestingly, the percentage of bound targets was significantly reduced following treatment with H and P, suggesting that compounds can interfere with selective dynamics of RNA recognition, with potential competition on purine-rich sequences at the intracellular level. The property of GAPDH as an AU-rich RNA-binding protein [53, 54] makes this observation more relevant in terms of specificity, since the treatment with compounds significantly impacted the general fraction of RNA bound by hnRNPA2B1 and TDP-43 but not the fraction bound by GAPDH, as evaluated by PY staining on immunoprecipitated protein/RNA-bead complexes (Supplementary Fig. S4B). In parallel, these results are consistent with experiments in Fig. 2D showing no interaction of hnRNPA2B1 with AU-rich RNA sequences.

Subsequently, we interrogated the EV-RNA keeping a 6 h treatment schedule with hit compounds. After incubation, we collected cells and recovered EVs for RNA isolation. Compounds induced a slight accumulation of intracellular RNAs that was significantly higher in the case of H and P (Supplementary Fig. S4C, left). In parallel, EV-RNA yields appeared highly variable (Figs. 4D and Supplementary Fig. S4C, right). To probe the quality of secreted EV-RNA, we elected miR-221 as a model readout, since it represented an abundant EV-transcript released from different cell types [55, 56], correlated with hnRNPA2B1 dosage [57], and reported as a substrate of several hnRNP members [58]. Interestingly, miR-221 was associated with exosome-mediated tumor phenotypes such as metastasis of cervical squamous carcinoma [59], malignancy of osteosarcoma cells [60], and paracrine effects in breast [61, 62] or bladder tumor cells [63]. In addition, miR-221 was one of the inflammatory miRNAs up-regulated in muscles of ALS patients [64] and a blood-circulating target that positively correlated with progression rate in sporadic ALS patients [65].

Treatment with compounds MH, TD, H, and P (but not ATA) induced a vesicular depletion of miR-221, even after normalization of the miRNA copy number against the endogenous Y3 RNA (Figs. 4E and Supplementary Fig. S4C). To validate these notions in other cellular models, we analysed miR-221 distribution in DU-145 prostate cancer cell lines and derived EVs, in this case, isolated by the exoEASY kit to diversify the analytical approaches on EV-RNA recovering. Supplementary Fig. S4D shows the distribution of miR-221 in cells and EVs following compound treatment, confirming the bioactivity of the small molecules tested. To substantiate the activity of compounds in a different cellular model, we also used human iPSC-derived motor neurons [66]. We grew iPSC-derived human smNPCs and differentiated them for 24 days to obtain mature motor neurons characterized by Islet1 and ChAt positivity [27] (Supplementary Fig. S5A). We treated differentiated cells for 6 h with compounds and all of them, except ATA in these cells, were able to induce a significant down-regulation of secreted EV-miR-221/Y3 copy number compared to DMSO (Fig. 4F).

In summary, selected bioactive drugs showed a coherent protein–RNA inhibitory activity, encompassing the journey from the biochemical experiments to cell-secreted EV-RNA, demonstrating that post-transcriptional regulation impacts the distribution of at least EXO-motif-bearing miRNAs and, therefore, the quality of EV-RNA sorting.

### Hematein and phenothrin counteracted the EV-induced paracrine activation of NF-kB

**Figure 5.**
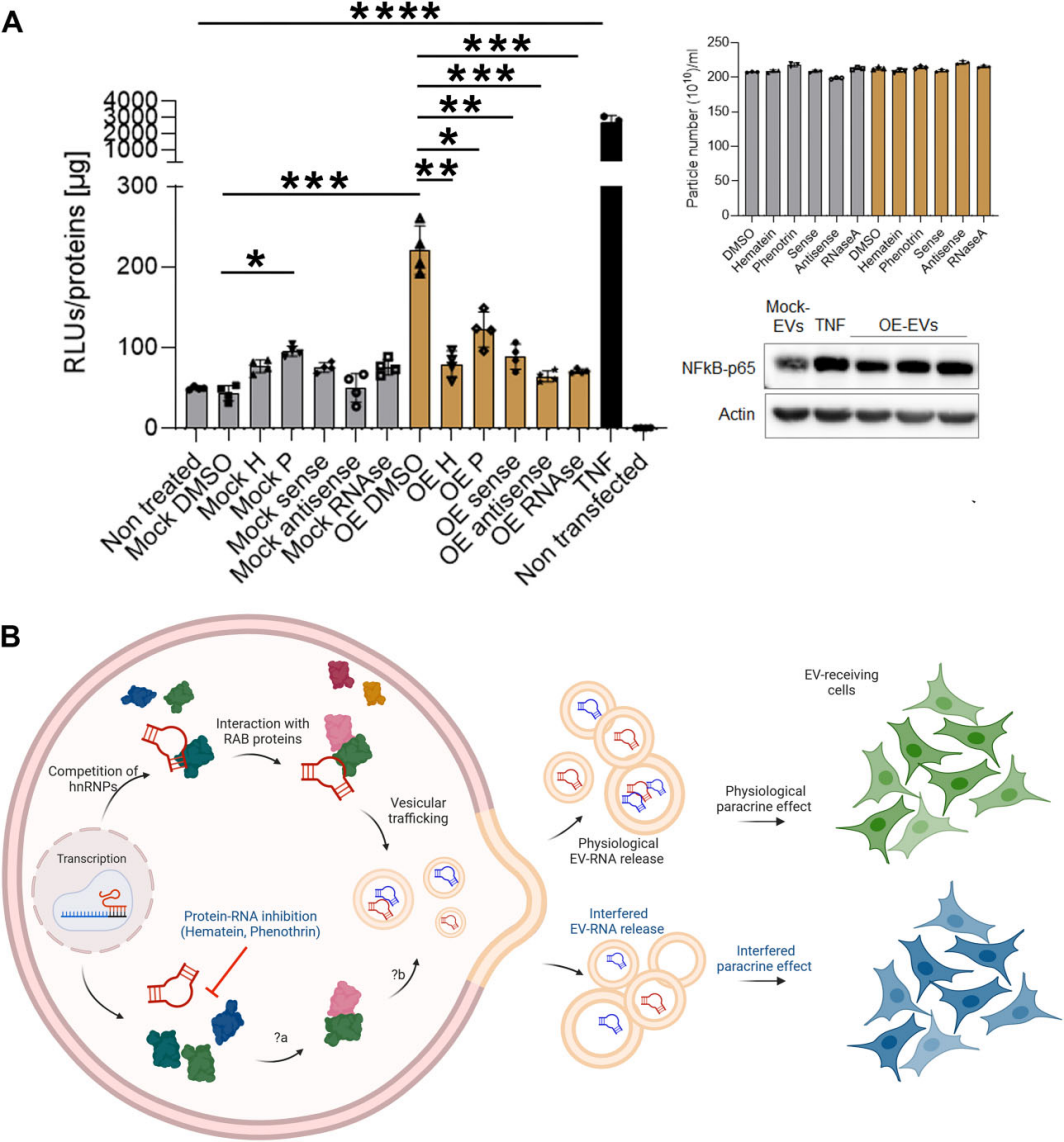
Paracrine effect on NF-kB activation of EVs deriving from RBP-conditioned and treated cells. (**A**) NF-kB activation in HEK293T upon acute treatment with EVs recovered from Mock or recombinant hnRNPA2B1-expressing cells (OE). Firefly luminescence was normalized on cellular protein content at the end point on the corresponding sample. Histogram reports mean and data of four independent experiments. The concentration of TNFα was 10 ng/ml. The concentration of RNAse A in the media was 2 μg/ml. Particles used to treat HEK293T cells were characterized by NTA (shown in Supplementary Fig. 5C) and the corresponding diameter is reported in the top right side. To have indication of NF-kB activation in EV-recipient cells, we performed an immunoblot using an anti-NF-kB antibody. *P-value < .05; ***P-value < .001; ****P-value < .0001. (**B**) Post-transcriptional control of EV RNA. Schematic representation of cytoplasmic events conveying RNA subsets into secreted EVs. RBPs, and in particular hnRNPs, could compete for and select RNAs. Interactions with other *trans*-acting factors, virtually RAB proteins participating in vesicular trafficking dynamics, could contribute to finalizing the RNA sorting process. Small molecule inhibitors of protein–RNA interactions, such as hematein or phenothrin, could alter intracellular RNA homeostasis and, ultimately, the distribution of the secreted counterpart, impacting paracrine functions. Direct consequences following the RNA recognition interference (?a) and vesicular packaging (?b) are still unclear. Created in BioRender. D’agostino, V. (2025) https://BioRender.com/h82u817.

To obtain some preliminary information on the bioactivity of identified compounds in relation to EV function, we explored the potential activation NF-kB in EV-recipient cells as a readout. Secreted miR-221, for example, was several times reported as associated with NF-kB activation in different epithelial cell lines [67–70]. We, therefore, used HEK293T cells as EV-recipient cells upon transfecting them with an NF-kB-responsive promoter activating the luciferase reporter. To work with an experimental set up considering the post-transcriptional effects on the readout, we evaluated if the expression of the recombinant hnRNPA2B1 could induce secretion of EVs capable of activating NF-kB if further exposed to autologous cells. Preliminary experiments in this sense demonstrated that the acute treatment with EVs secreted by hnRNPA2B1-OE cells could cause a significant induction of NF-kB responsive promoter. We, therefore, exploited this system to test compounds including some indication on the relevance of the EV-RNA cargo in mediating the luciferase activation in recipient cells. We transfected HEK293T cells with Mock or hnRNPA2B1 (OE) plasmid and 24 h later we treated cells with 5 μM of hematein and phenotrin compounds for 6 h. Next, we washed with PBS and incubated cells with serum-free media for additional 24 h to recover EVs. We exposed these EVs for 8 h to HEK293T cells previously seeded in 96-well plate and transfected with the luciferase reporter plasmid for 24 h. Remarkably, the expression of the hnRNPA2B1 protein correlated with secretion of EVs able to induce a significant activation of NF-kB, although with a lesser extent if compared to 10 ng/ml of TNFα added as positive control (Fig. 5A). In these settings, both compounds (H and P) significantly counteracted the EV-mediated, hnRNPA2B1-induced NF-kB activation, confirming the expected outcomes. Interestingly, treatment with phenotrin in Mock cells caused a slight, but significant increase of luciferase signal, possibly indicating promiscuous/parallel mechanisms of action of this compound. However, these results did not provide direct correlation with the EV-RNA quality. Therefore, we performed these experiments including EVs deriving from HEK293T cells transfected with sense and antisense probes with the idea of interfering with the pool of secreted EV-RNA in turn recognized in recipient cells. Moreover, we included EVs sedimented by UC after incubation of media with 2 μg/ml RNAse A for 30 min. The two RNA probes surprisingly counteracted the hnRNPA2B1-mediated effects on secreted EVs, rescuing the luciferase activation to Mock levels, an outcome requiring deeper knowledge of the mechanism behind. Interestingly, the RNAse treatment led to EVs that lost such effects observed by concomitant hnRNPA2B1 expression (Fig. 5A). NTA profiling of the EVs used in these experiments (Supplementary Fig. S5B) indicated no significant variations in particles concentration; the slight fluctuations in mean diameter are reported in Fig. 5A, top right. We found this last observation particularly interesting because we cannot exclude that sedimented particles are completely free of enzyme, therefore underestimating the effect we observed. In these settings, NTA performed on the particles used indicated no significant changes in concentration with the fluctuations in mean diameters shown in Fig. 5A, top right. In line with the purpose of this study, these data indicated the involvement of EV-RNA in mediating NF-kB effects in recipient cells and the relevance of hnRNPA2B1-mediated post-transcriptional function to this paracrine activity. To substantiate the NF-kB activation in recipient cells, we performed an immunoblot showing the effective upregulation of this protein in total cell lysates (Fig. 5A, bottom right). These results suggested that the activity of specific RBPs can condition the secretome and, in turn, influence the NF-kB response in recipient cells, as schematized in Fig. 5B.

In conclusion, this work demonstrates that EV-RNA secretion is a dynamic circuit composed of post-transcriptional *cis/trans*-acting factors that small molecules could selectively interfere to challenge EV quality and discrete paracrine functions.

## Discussion

In this work, we designed a new biochemical approach to capture cumulative interactions between RBPs and secreted EV-RNA *in vitro*, assuming the “binding” as an exemplified proxy of RNA sorting involvement. Several RBPs have been recently described as part of the EV protein cargo responsible for sorting different RNA species [6]. Since hnRNPA2B1 gained visibility in this process, we considered this protein the first candidate in our perturbation experiments, exploiting the protein enrichment/depletion as a direct surrogate of affinity/competition on the EV-RNA probe. Interestingly, a recombinant hnRNPA2B1 protein representing only 25% of the endogenous protein dosage was enough to detect competition effects, providing evidence of a certain degree of obtained sensitivity or protein binding efficiency, given hnRNPA2B1 a central node in the protein network we pulled down. Despite limitations due to a mixture of heterogeneous RNA fragments, which could generate sub-optimal RNA-binding/protein capture, and that not all the emerged protein–RNA interactions may necessarily be engaged in EV-RNA sorting pathways, the reverse proteomics approach indicated functional connections between selected hnRNPs and RAB proteins (at least responding to ectopic hnRNPA2B1 expression) over more than 2000 proteins identified by MS. hnRNP members such as hnRNPA2B1 or hnRNPA1 were already described in regulatory networks together with RAB members [71]. Moreover, two ranked hnRNPs (TDP43 and hnRNPD) were already described in direct or functional associations with hnRNPA2B1 [72, 73], suggesting possible critical players in concomitant mechanisms of EV-RNA selection and EV biogenesis, yet unclarified. In perspective, these data acquire more relevance if considering the role of TDP43 as potential biomarker for monitoring disease progression of frontotemporal dementia and ALS, as recently demonstrated [74, 75], and get more insights on the quality of vesicles secreted in biofluids in comparison to cell-derived ones.

Although reverse proteomics did not directly inform on the quality of bound RNA sequences, we could prioritize purine-rich sequences by merging the association with hnRNP binders and experimentally validated sequence substrates. Interestingly, the purine-rich RNA was one of the previously identified EXO motifs and one of the most enriched sequences in EV-transcriptomic analyses upon protein silencing strengthening our inference. Importantly, we present here a new tool based on short-length, fluorescent RNA oligos to probe the quality of secreted EV-RNA, confirming the relevance of the sequence motif. Further investigation on different cell and EV sources is required to systematically address the relative proportion of secreted RNA sequences. Since TDP43, hnRNPUL2, hnRNPD, and hnRNPA2B1 itself share a common purine-rich RNA recognition, we exploited this notion to set up a platform for challenging RBP–RNA interactions anchoring on hnRNPA2B1 activity and explore the possibility of interfering with the vesicular distribution of selected transcripts.

We searched for the best strategy to challenge a full-length hnRNPA2B1 protein, detected as EV cargo [6]. Unlike the first two common RNA recognition motifs, we argued that a full-length protein could behave intracellularly with an oligomerization capacity, virtually affecting the local or general RNA-binding dynamics. Our pilot screening campaign probed small molecules that might present some structural analogies and converge on the inhibition of hnRNPA2B1-EXO-motif RNA interactions. These compounds could biochemically interfere with direct RNA recognition without excluding possible anti-oligomerization effects on hnRNPA2B1. We provide evidence that at least two hit compounds could have RNA-binding selective and/or combined effects in sub-cellular contexts, justifying the observed variable effects on secreted EV-RNA.

The short-term treatment we applied represents a suboptimal schedule not individually tailored to the different cells tested, as we wanted to explore post-transcriptional dynamics, minimizing other secondary events related to cellular stress. In fact, we treated cells with a compound concentration (5 μM) which was far from inducing cell death in NSC34 or HEK293T cells (Supplementary Fig. S6) or stress-related inclusions impacting the sub-cellular localization of hnRNPA2B1 protein (Supplementary Fig. S7). Besides their targeting surfaces, the heterogeneous activity of the identified compounds could potentially feature different cell permeability and association rates/modes. The notions we collected here regarding the *in vitro* RNA-binding properties of hematein and phenothrin, do not restrict RNA as the only surface interaction of these small molecules. Hematein, for example, has been reported as a protein kinase CK2 inhibitor [76], while phenothrin is a synthetic DNA-damaging insecticide [77]. Notwithstanding, these compounds could preferentially impact post-transcriptional control and vesicular RNA sorting. These observations could suggest these small molecules as potential new scaffolds in medicinal chemistry to prioritize interactions with RNAs. At this point, other fascinating questions warrant further investigation on other RNA species containing purine-rich motifs, the potential influence of post-transcriptional RNA modifications, and the parallel post-translational modifications of hnRNPs [25, 26].

Since EXO-motif RNA sequences were shown to characterize a plethora of pri- and mature miRNAs, we chose miR-221 as a surrogate readout of EV-RNA fluctuations and a functional target with an established link with pro-inflammation processes. Interestingly, hnRNPA2B1 [78] and miR-221–3p [79] were individually associated with inflammation. In line with biochemical findings, we could observe a consistent reduction of EV-miR-221 packaging upon short-lasting treatment of different cells. To emphasize the role on post-transcriptional regulation and exclude that compounds themselves could have direct anti-inflammatory effects on EV recipient cells, EV donor cells were shortly treated with a low micromolar range of compounds. After the incubation, cells were extensively washed with PBS and then incubated with serum-free media to recover EVs. This treatment strategy was designed to avoid direct exposure of EV receiving cells to free compounds, not considering a potential small molecule/metabolite fraction eventually released in the medium by the cells through EVs. Unfortunately, we did not find exploitable compound ions that we could apply for MS-based detection. Nevertheless, we found a consistent counteracting effects of tested compounds mediated by EVs secreted from hnRNPA2B1 expressing condition. The mechanism underlying the association between the EV biological content and the paracrine NF-kB activation still remains to be addressed, but we observed a strong reduction of the pro-inflammatory effect after treating EVs with RNAse A. We cannot exclude the activation of pro-inflammatory pathways either contributed by EV surfaces or RNA-independent mechanisms, nor the potential effects of extracellularly secreted protein–RNA complexes. In this regard, a set of dedicated experimental strategies is needed to dissect the quantitative contribution of RNA to this signaling, also expanding the repertoire of cells under testing and size of cargo vesicles. The reduction of miR-671–5p in EVs from menstrual blood-derived stem cells is responsible for the positive regulation of NF-kB [80] or that EVs deriving from senescent cells and enriched in miR-30b-5p induce IL-1β and IL-6 interleukins with concomitant activation of NF-kB pathways [81], or that EVs shuttling miR-660 promote breast cancer progression through a KLHL21-mediated IKKβ/NF-kB p65 axis [82] are other observations in line with an RNA-induced effect in EV recipient cells. Consistently, hit compounds interfered with EXO RNA recognition and impacted EV-secreted RNA/quality, counteracting the hnRNPA2B1-induced NF-kB activation irrespective of the number of particles released. For example, this activation is considered pathogenic in ALS [83, 84], and we provide evidence that secreted EV-RNAs can mediate this paracrine effect. We demonstrate here that a substantial post-transcriptional control impacts selected EV-RNA secretion, and interfering with the underlying protein–RNA interactions can be instrumental in modulating the EV-associated paracrine physiology.

We open a new scenario where the simultaneous manipulation of RBPs and RNA harboring specific consensus sequences could be explored to identify new strategies for targeting paracrine functions.

## Supplementary Material

gkaf176_Supplemental_Files

## Data Availability

MS analyses and raw data generated in this study are included in Supplementary material and online (PRIDE, https://www.ebi.ac.uk/pride/, project accession: PXD052658).
