# Supplementary material for: Small molecule inhibitors of hnRNPA2B1–RNA interactions reveal a predictable sorting of RNA subsets into extracellular vesicles
Source: Nucleic Acids Res. 2025 Mar 18;53(5):gkaf176. doi: 10.1093/nar/gkaf176 (PMC11915509; doi:10.1093/nar/gkaf176)
Supplement: gkaf176_Supplemental_Files [file gkaf176_supplemental_files.zip › Supplementary Files REV_2.docx]

**SUPPLEMENTARY TABLES, FIGURES, AND FIGURE LEGENDS**

**Supplementary Table 1.** MS of detected proteins and analysis of hnRNPA2B1 OE versus Mock condition.

**Supplementary Table 2.** RAB proteins resulting from hnRNPA2B1 OE versus Mock condition.

**Supplementary Table 3.** MS of detected proteins and analysis of TDP43 OE versus Mock condition.

**Supplementary Figure S1**

**Supplementary Figure S1**. **Ontologies and RNA consensus motifs of hnRNPs identified by reverse proteomics**. **(A)** Gene Ontology (GO) analysis of proteins enriched (up) or displaced (down) in the ratio between hnRNPA2B1 OE and Mock conditions. The dimension of the point is given by the Odds Ratio in log2 scale. The filling is based on the significance of the adj. *p* value against the selected threshold. **(B)** Interaction network inside the 336 enriched protein targets obtained from STRING database (<https://string-db.org>). **(C)** Volcano Plot representing the differentially expressed proteins as described in Figure 1 but using lysates from TDP43-expressing cells. Raw data are available in Table S3. **(D)** Experimentally validated RNA sequence motifs reported as recognized by the identified hnRNPs Sequence motifs (<http://rbpdb.ccbr.utoronto.ca>) and analysed by MEME toolbox (<https://meme-suite.org/meme>).

**Supplementary Figure S2**

**Supplementary Figure S2. Synthetic RNA, GST-hnRNPA2B1 protein purification, and AlphaScreen titration**. **(A)** RNA secondary structure prediction of the 5’-GGGGAGGUUAGGGAGGAGGGGGGUAGGCGCC sequence using RNAstructure (<https://rna.urmc.rochester.edu>), as calculated with the lowest (-0.5 kcal/mol) free energy. **(B)** Left: Coomassie of GST-hnRNPA2B1 purification. GST-hnRNPA2B1 concentration was calculated using 1 μg of gel-loaded BSA as reference. Right: A280 nm absorbance profile of a representative eluate indicating purity of purified recombinant protein. The obtained protein concentration was ~2 µM on average according to three independent experiments here reported. **(C)** Western blot using an anti-hnRNPA2B1 antibody and confirming the purification of the protein. “NI” non-induced bacteria; “I”, IPTG- induced bacteria, “W1, W2”, Wash 1 and 2; “EL”, elutions. **(D)** Graph showing the hooking point of the protein, reached at 30 nM. Hill coefficient was equal to 0.3 as calculated by nonlinear regression using specific binding with Hill slope fitting model of GraphPad Prism 9. Mean and standard deviations refer to three independent measurements.

**Supplementary Figure S3**

**Supplementary Figure S3. Fluorescence intensity displacement (FID).** Purine-rich and ARE RNA probes were used for melting curve profiling using Midori green fluorescence in the presence of P and H compounds. Measurements were performed on a BIORAD CFX384 instrument.

**Supplementary Figure S4**

**Supplementary Figure S4. EV-RNA analysis upon hnRNPA2B1 ectopic expression or silencing and compound treatment**. **(A)** Left: Representative NTA profiles of EVs retrieved after hnRNPA2B1 overexpression or silencing. Right: Representative Bioanalyzer profile of EV-RNA retrieved after hnRNPA2B1 expression or silencing. **(B)** Flow cytometry experiments to evaluate the global RNA staining of RNA-immunoprecipitated/bead complexes representing a fraction of RIP samples (Figure 4). RNA staining was obtained using RNA/protein-antibody-bead complexes, was performed on the same instrument using Pyronin Y (PY) was used at a final concentration of 50 ng/ml and the PY-positive events recorded for 1 min. Sequential gatings with Streptavidin beads alone +/- antibodies was used to detect the specific signal. Mean and SD refer to three independent measurements corresponding to the RIP samples analysed in Figure 4. **(C)** Nanodrop quantification of total RNA isolated from a total of 10^6^ cells (histogram on the left) and EV-RNA profiles of corresponding cells treated for 6 hr with the indicated compounds. Statistical analysis was performed using three independent experiments; * indicates p value <0.05. **(D)** miR-221 quantification using DU-145 cells and derived EVs. Left: ddPCR assay of miR-221 detection from DU-145-derived RNA; Center: miR-221 detection in EV-RNA collected from DU-145 EVs; Right: Ratio between values obtained in cells and EVs. Graphs report data of two independent experiments.

**Supplementary Figure S5**

**Supplementary Figure S5. Characterization of iPSC-derived motor neurons. (A)** Representative brightfield image of 10 days-differentiated and 2 weeks-matured motor neurons. **(B)** Immunofluorescence staining of iPSC-derived motor neurons after final differentiation. Left: pan neuronal marker microtubule associated protein tau (MAP2), mature lower motor neuron marker choline acetyltransferase (ChAt), total cell nuclei marker Hoechst. Right: Early motor neuron marker transcription factor ISELT1, mature lower motor neuron marker choline acetyltransferase (ChAt), total cell nuclei marker Hoechst. Magnification 20x. **(C)** Nanoparticle tracking analysis of EVs recovered from HEK293T cells transfected with no DNA (Mock) or hnRNPA2B1 (OE) plasmid, either treated with DMSO or H and P compounds, or with 2 µg/ml RNAse A for 30 min at RT before subjecting samples to differential ultracentrifugation as described in Figure 5.

**Supplementary Figure S6**

**Supplementary Figure S6. Cell viability upon treatment with Hematein.** Cells were exposed to increasing concentrations of Hematein or DMSO (control vehicle) for 24 hr under standard growth conditions. The dose response curves indicated Hematein IC_50_ of ~78 µM with NSC34 cells and ~41 with HEK293T cells. In both cases a range of drug tolerance was observed before the drop of viability over 10 µM. The graph indicates mean and SD of two independent experiments.

**Supplementary Figure S7**

**Supplementary Figure S7. Immunofluorescence of hnRNPA2B1.** NSC34 cells were washed twice with PBS without Ca2+/Mg2+ (LifeTechnologies) and fixed with 4% PFA in PBS for 10 min at room temperature, afterwards washed three times with PBS. Fixed cells were permeabilized for 10 minutes in 0.2 % Triton X solution and then incubated for 1 hour at RT in blocking solution (1% BSA, 5% donkey serum, 0.3M glycine and 0.02% Triton X in PBS). Primary antibodies were diluted in blocking solution and cells were incubated with this solution overnight at 4°C. Alexa-594-conjugated mouse secondary antibody was used to recognize the target. Cells were also incubated with 10 ng/ml Hoechst before acquiring images with a Zeiss confocal microscopy.
